# Maize Tolerance against Drought and Chilling Stresses Varied with Root Morphology and Antioxidative Defense System

**DOI:** 10.3390/plants9060720

**Published:** 2020-06-06

**Authors:** Hafiz Athar Hussain, Shengnan Men, Saddam Hussain, Qingwen Zhang, Umair Ashraf, Shakeel Ahmad Anjum, Iftikhar Ali, Longchang Wang

**Affiliations:** 1 College of Agronomy and Biotechnology, Southwest University/Engineering Research Center of South Upland Agriculture, Ministry of Education, Chongqing 400716, China; atharhussainswu@yahoo.com (H.A.H.); men0625@163.com (S.M.); iftikharaliswu@yahoo.com (I.A.); 2 Institute of Environment and Sustainable Development in Agriculture, Chinese Academy of Agricultural Sciences, Beijing 100081, China; zhangqingwen@caas.cn; 3 Department of Agronomy, University of Agriculture, Faisalabad 38040, Pakistan; shakeelanjum1034@gmail.com; 4 Department of Botany, Division of Science and Technology, University of Education, Lahore 54770, Pakistan; umair.ashraf@ue.edu.pk

**Keywords:** maize, antioxidant defense system, osmolyte, chilling, drought, reactive oxygen species

## Abstract

Maize belongs to a tropical environment and is extremely sensitive to drought and chilling stress, particularly at early developmental stages. The present study investigated the individual and combined effects of drought (15% PEG-Solution) and chilling stress (15/12 °C) on morpho-physiological growth, osmolyte accumulation, production of reactive oxygen species (ROS), and activities/levels of enzymatic and non-enzymatic antioxidants in two maize hybrids (i.e., “XD889” and “XD319”) and two inbred cultivars (i.e., “Yu13” and “Yu37”). Results revealed that individual and combined exposure of drought and chilling stresses hampered the morpho-physiological growth and oxidative status of maize cultivars, nevertheless, the interactive damage caused by drought + chilling was found to be more severe for all the studied traits. Between two individual stress factors, chilling-induced reductions in seedling length and biomass of maize cultivars were more compared with drought stress alone. Greater decrease in root length and biomass under chilling stress ultimately decreased the volume and surface area of the root system, and restricted the shoot growth. All the stress treatments, particularly chilling and drought + chilling, triggered the oxidative stress by higher accumulation of superoxide anion, hydrogen peroxide, hydroxyl ion, and malondialdehyde contents compared with the control. Variations in response of maize cultivars were also apparent against different stress treatments, and XD889 performed comparatively better than the rest of the cultivars. The better growth and greater stress tolerance of this cultivar was attributed to the vigorous root system architecture, as indicated by higher root biomass, root surface area, and root volume under drought and chilling stresses. Moreover, efficient antioxidant defense system in terms of higher total antioxidant capability, superoxide dismutase, peroxidase, catalase, and glutathione reductase activities also contributed in greater stress tolerance of XD889 over other cultivars.

## 1. Introduction

Crop plants often experience multiple abiotic stresses simultaneously, of which concurrent effects of sub-optimal temperature and moisture deficit conditions are perhaps the most deleterious for plant growth [1,2,3]. Drought and chilling stresses cause substantial reductions in growth and yield attributes of plants by disturbing normal plant metabolism [2,4]. Sub-optimal temperature causes water deficiency in maize seedlings by substantial reduction in root water uptake coupled with leaves transpiration [5]. Likewise, drought stress induces the stomatal closure and decline the transpiration in maize [6]. Hussain et al. (2018) concluded that the both drought and chilling stresses disrupt the plant–water relations, but these effects are possibly more detrimental under the simultaneous occurrence of these both factors [2]. However, the effects of these stress factors depend on the duration, nature, and severity of stress and the plant developmental stages. Chilling stress was found to decrease the root growth of maize seedlings by decreasing the length and biomass of the roots [7], in contrast, drought stress increased the root length of sunflower plants but decreased later on with an increase in drought period [8]. Previous investigations have shown that the osmotic balance of the plants is more affected by drought as compared to chilling stress [9].

Generally, the effects of both these stresses on most of the plant morphological traits are quite similar; however, their mechanisms to affect the physio-biochemical processes of plants may vary [10]. Most often, individual and/or concurrent drought and chilling stresses result in excessive production of reactive oxygen species (ROS) that cause structural and functional damage to the cell and its constituents [2,11], whereas the degree of oxidative damage may be accelerated synergistically under combined drought and chilling stresses [12,13,14]. In order to control the overproduction of ROS under different abiotic stresses, the plants have a highly efficient and sophisticated enzymatic and non-enzymatic antioxidative defense system [15,16,17]. It has been well reported that drought and chilling tolerance in plants is strongly linked with the enhanced activities/levels of antioxidants under stress condition [18,19,20]. Along with antioxidants, accumulation of different osmolytes (such as proline, proteins, carbohydrates) also play an important role in stress tolerance through maintenance of turgor and protection of macromolecules in dehydrating plants cells [17].

Maize is a thermophilic crop and belongs to a tropical environment with optimum growth temperature around 28 °C [21]. Nevertheless, at emergence and early growth stages, the maize seedlings are extremely sensitive to drought and low temperature. Water deficiency and sub-optimal temperatures (<12–15 °C) during seedling growth can be detrimental to subsequent crop productivity [2,6]. Although the inhibitory effects of sub-optimal temperature and low moisture supply on morpho-physiological growth and productivity of various crops have been reported previously [20,22,23,24]; however, little information is available about the individual and concurrent effect of these stressors on morpho-physiological growth, root system architecture, and oxidative metabolism in hybrid and inbred maize cultivars. It was hypothesized that the individual and combined drought and chilling stresses will differentially affect the growth, root system architecture, oxidative metabolism, and osmolyte accumulation of maize, and the response of maize hybrid and inbred cultivars will be different to these stress factors. Therefore, the present study was conducted, (1) to assess the effect of chilling and drought stress applied individually or in combination on the morpho-physiological growth, root morphology, and oxidative metabolism of inbred and hybrid maize cultivars; and (2) to explore the possible mechanism/s of maize tolerance against drought and chilling stresses. 

## 2. Results

### 2.1. Shoot Growth

**Figure 1:**
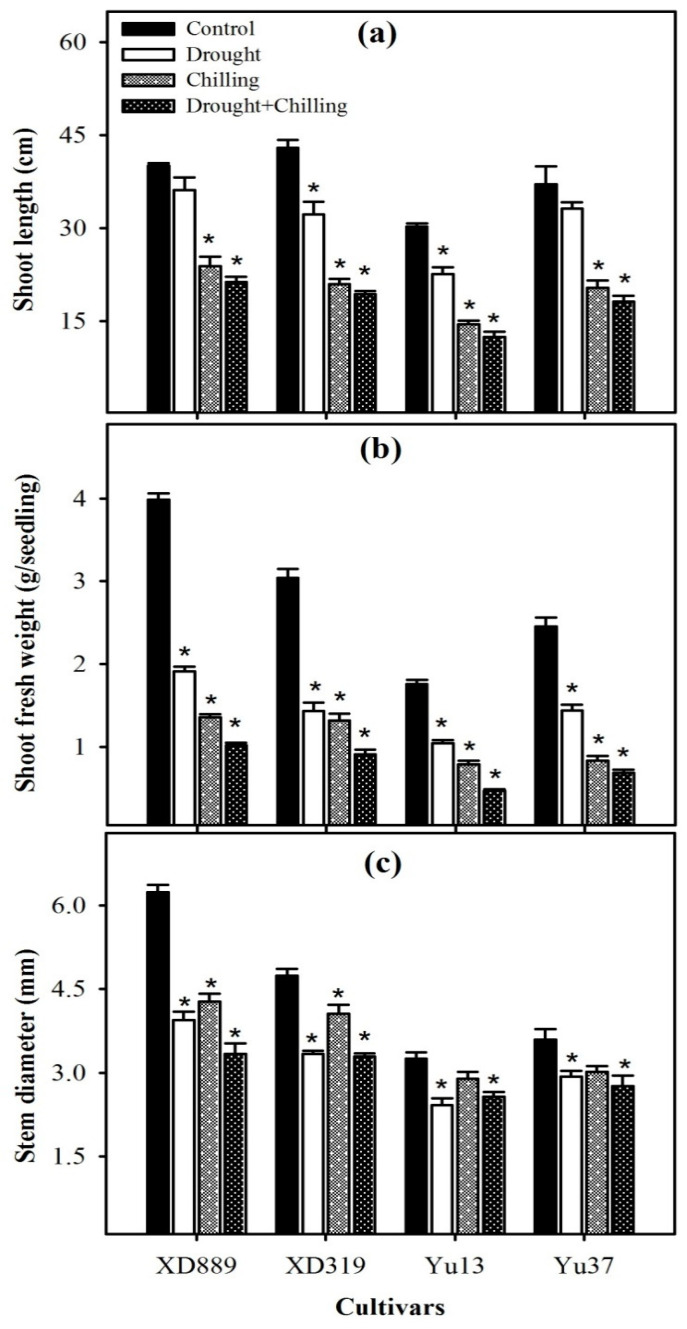
Shoot length (**a**), shoot fresh weight (**b**), and stem diameter (**c**) of four maize cultivars as influenced by drought, chilling, and drought + chilling stress. Vertical bars above mean indicate standard error of three replicates. Mean value for each treatment with * indicate significant differences compared with control according to least significant difference (LSD) test (*p* ≤ 0.05).

**Figure 2:**
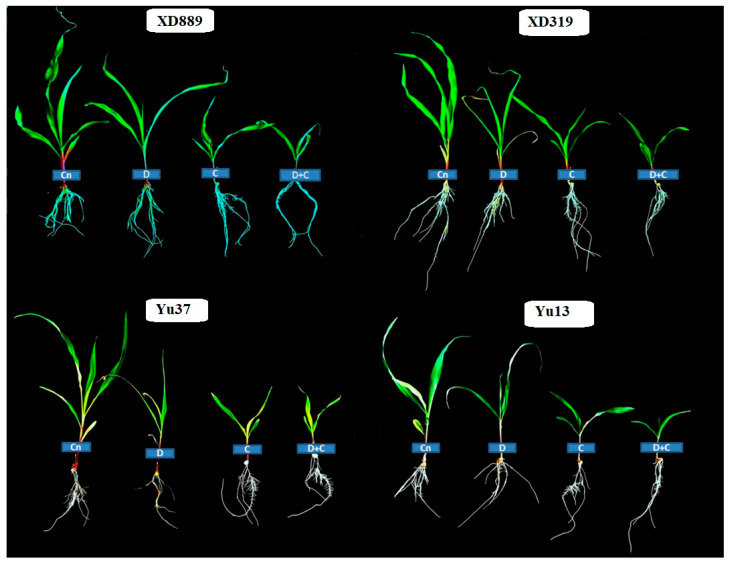
Individual and interactive effect of drought and chilling stresses on the seedlings growth of maize (cv. XD889, XD319, Yu13, Yu37). Cn—control, D—drought, C—chilling, D + C—drought + chilling.

Pronounced variations were observed in shoot growth of all maize cultivars under the influence of drought, chilling, and drought + chilling stresses (Figure 1 and Figure 2). Compared with the control, the shoot length of all maize cultivars was significantly (*p* < 0.05) reduced by chilling alone and drought + chilling stresses, nevertheless, the negative effects of drought stress alone on shoot length were only significant for XD319 and Yu13. All the stress treatments significantly (*p* < 0.05) reduced the shoot fresh weight and stem diameter of maize cultivars compared with the control, except the stem diameter of Yu13 and Yu37 was unaffected by chilling stress. The negative effects of combined drought + chilling stress were more severe for all these shoot growth attributes compared with their individual effects (Figure 1). The shoot growth performance of XD889 was comparatively better than other maize cultivars under control as well as stress conditions (Figure 1). 

### 2.2. Leaf Growth

**Figure 3:**
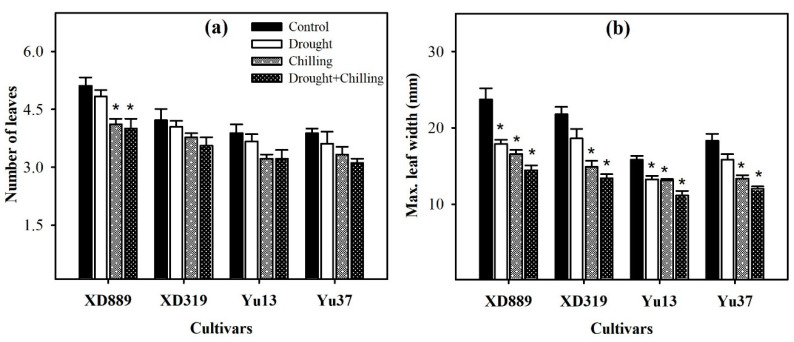
Number of leaves (**a**) and maximum leaf width (**b**) of four maize cultivars as influenced by the drought, chilling, and drought + chilling stresses. Vertical bars above mean indicate standard error of three replicates. Mean value for each treatment with * indicate significant differences compared with control according to LSD test (*p* ≤ 0.05).

Pronounced reductions in number of leaves and maximum leaf width of all maize were observed under the influence of drought, chilling, and drought + chilling stresses (Figure 3); however, the effects of stress treatments were variable. The number of leaves in XD889 was only reduced significantly (*p* < 0.05) under chilling and drought + chilling stress, compared with the control. Whereas all the stress treatments significantly (*p* < 0.05) decreased the maximum leaf width of all the maize cultivars except for XD319 and Yu37 under drought stress (Figure 3). The XD889 recorded a comparatively higher number of leaves and maximum leaf width compared with the rest of the cultivars under control as well as stress conditions (Figure 3). 

### 2.3. Root Growth

**Figure 4:**
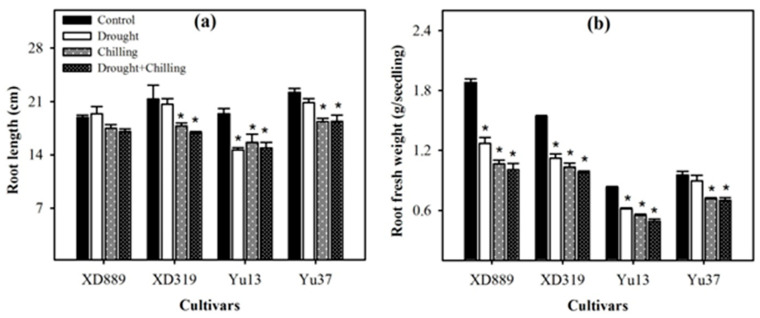
Root length (**a**) and root fresh weight (**b**) of four maize cultivars as influenced by the drought, chilling, and drought + chilling stresses. Vertical bars above mean indicate standard error of three replicates. Mean value for each treatment with * indicate significant differences compared with control according to LSD test (*p* ≤ 0.05).

**Figure 5:**
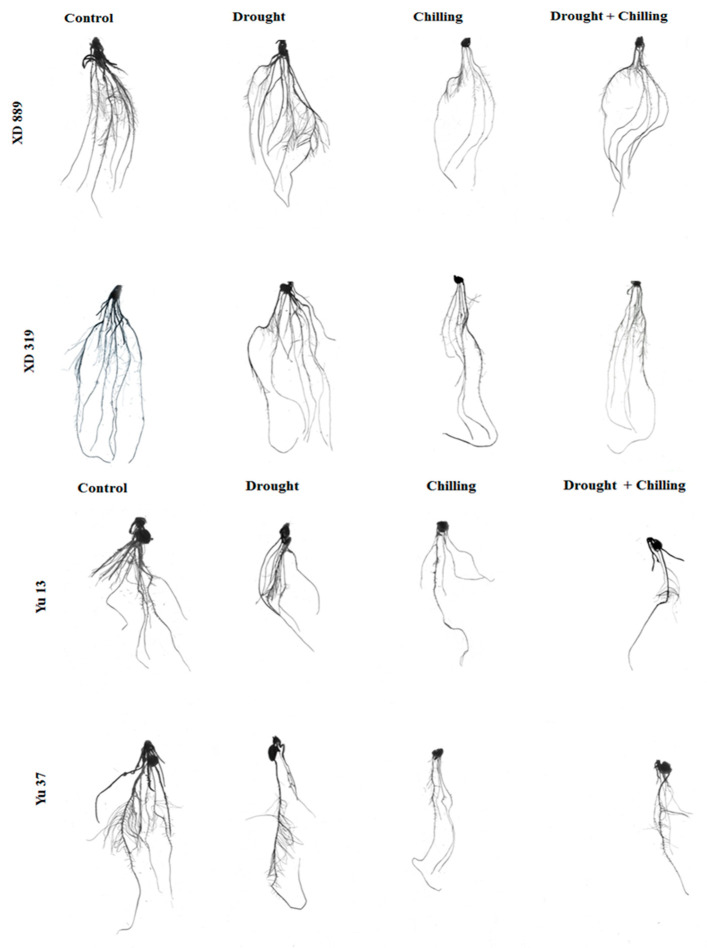
Root growth of maize seedlings (cv. XD889, XD319, Yu13, Yu37) under drought, chilling, and drought + chilling stress conditions.

**Figure 6:**
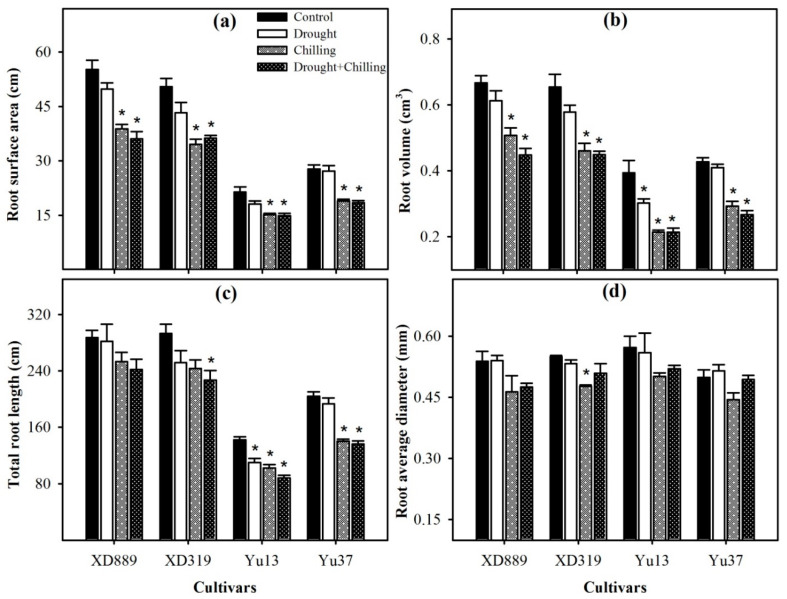
Root surface area (**a**), root volume (**b**), total root length (**c**), and root average diameter (**d**) of four maize cultivars as influenced by the drought, chilling, and drought + chilling stresses. Vertical bars above mean indicate standard error of three replicates. Mean value for each treatment with * indicate significant differences compared with control according to LSD test (*p* ≤ 0.05).

Individual and concurrent drought and chilling stress conditions severely hampered the root growth of maize cultivars (Figure 4, Figure 5 and Figure 6). Root length of XD889 was slightly increased under drought stress but reduced under chilling, and drought + chilling stress conditions, although, these effects remained statistically non-significant (*p* ˃ 0.05). The root lengths of XD319, Yu13, and Yu37 were significantly (*p* < 0.05) reduced under chilling, and drought + chilling stress as compared with control; the drought-induced reductions in root length were only significant for Yu13 (Figure 4a). Root fresh weight of all maize cultivars were decreased under all the stress treatments, the influence of drought stress on root fresh weight of Yu-37 was statistically non-significant (*p* ˃ 0.05) compared with the control (Figure 4b).

Pronounced reductions in root surface area, root volume, and total root length of all maize cultivars were noticed under different stresses; however, variations in response of these attributes were apparent among different stress treatments (Figure 6). Compared with the control, the root surface area and root volume of four maize cultivars were significantly (*p* < 0.05) decreased under chilling and drought + chilling stress. Drought stress alone did not significantly alter both these traits in all maize cultivar expect for root volume in Yu13. The XD889 recorded higher root surface area and root volume compared with all other cultivars. Moreover, the total root length and root average diameter in this cultivar under all stress treatments remained statistically similar (*p* > 0.05) to the control. Total root length in Yu13 was significantly (*p* < 0.05) decreased by all the stress treatments, while for Yu37, only chilling and drought + chilling stresses significantly decreased the total root length compared with the respective control. The root average diameter was only decreased significantly (*p* < 0.05) in XD319 under chilling stress over the control (Figure 6).

### 2.4. Photosynthetic Pigments

**Figure 7:**
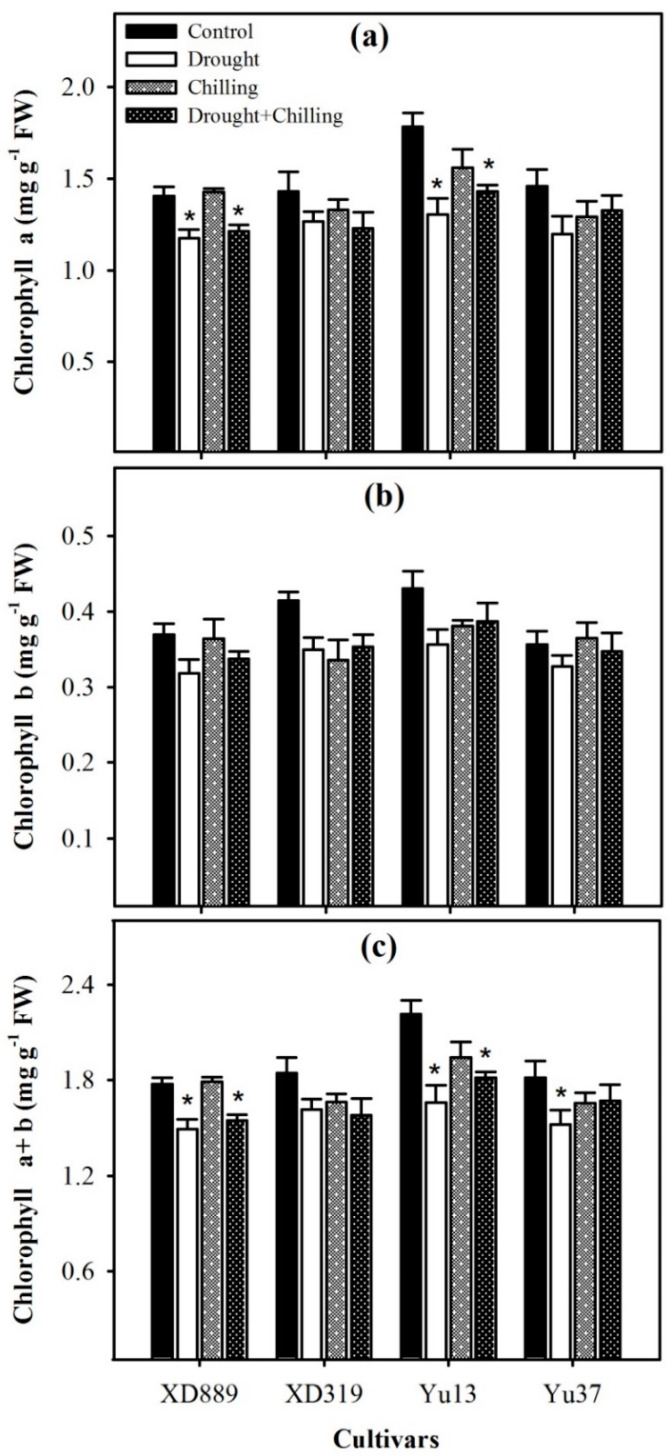
Chlorophyll a content (**a**), chlorophyll b content (**b**), and chlorophyll a + b content (**c**) in four maize cultivars as influenced by the drought, chilling, and drought + chilling stresses. Vertical bars above mean indicate standard error of three replicates. Mean value for each treatment with * indicate significant differences compared with control according to LSD test (*p* ≤ 0.05).

Chilling stress alone did not significantly (*p* > 0.05) affect the chlorophyll a, chlorophyll b, and chlorophyll a + b contents in all the maize cultivars compared with control, nevertheless, the impact of drought and drought + chilling stress on the chlorophyll a and chlorophyll a + b contents of XD 889 and Yu13 were significant (*p* < 0.05) as compared with the control (Figure 7a,c). For all the maize cultivars, the chlorophyll b contents under stress treatments were statistically similar (*p* ˃ 0.05) with the control (Figure 7b). 

### 2.5. Reactive Oxygen Species and Lipid Peroxidation 

**Figure 8:**
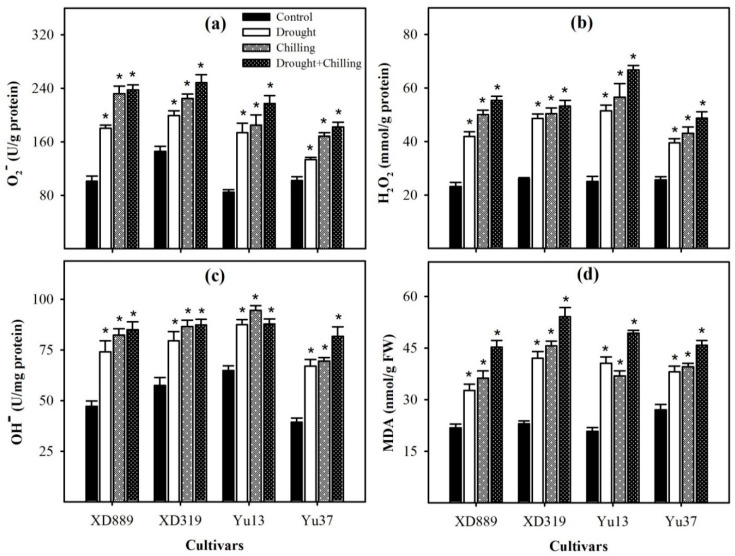
Accumulation of superoxide radical (**a**), hydrogen peroxide (**b**), hydroxyl ion (**c**), and malondialdehyde content (**d**) in the leaves of four maize cultivars as influenced by the drought, chilling, and drought + chilling stress. Vertical bars above mean indicate standard error of three replicates. Mean value for each treatment with * indicate significant differences by the LSD test (*p* ≤ 0.05).

Significant variations (*p* < 0.05) in ROS accumulation and lipid peroxidation rate in four maize cultivars were observed under the influence of drought, chilling, and drought + chilling stresses; however, differences were apparent among different stress treatments. Compared with the control, superoxide anion (O_2_^−^), hydrogen peroxide (H_2_O_2_), hydroxyl ion (OH^−^), and malondialdehyde (MDA) contents in all maize cultivars were significantly increased (*p* < 0.05) under individual and combined drought and chilling stresses (Figure 8). The maximum ROS concentrations and lipid peroxidation rate were recorded under combined drought + chilling stress. Among different cultivars, XD319 recorded the maximum MDA and O_2_^−^contents, while the highest H_2_O_2_ content was observed in Yu13 (Figure 8). 

### 2.6. Enzymatic Antioxidants

**Figure 9:**
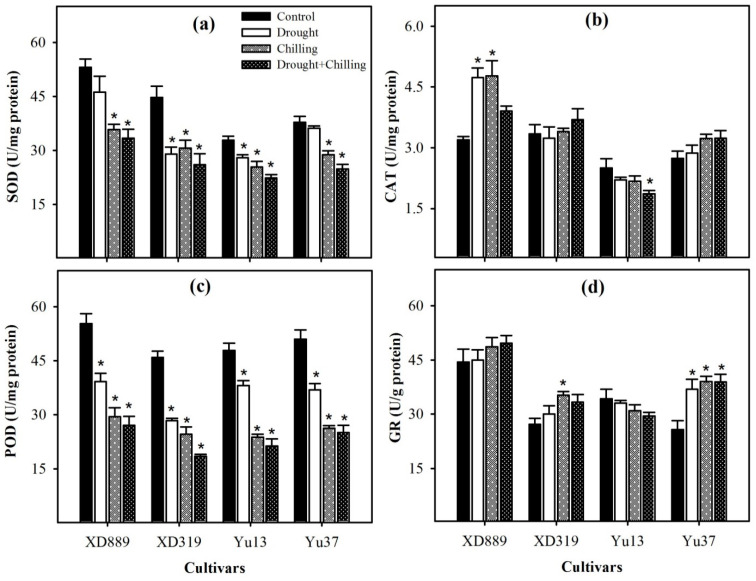
Activities of superoxide dismutase (**a**), catalase (**b**), peroxidase (**c**), and glutathione reductase (**d**) in the leaves of four maize cultivars as influenced by the drought, chilling, and drought + chilling stresses. Vertical bars above mean indicate standard error of three replicates. Mean value for each treatment with * indicate significant differences compared with control according to LSD test (*p* ≤ 0.05).

Superoxide dismutase (SOD) and peroxidase (POD) activities were significantly (*p* < 0.05) reduced in all maize cultivars under drought, chilling, and drought + chilling stress treatments; only SOD activities in XD889 and Yu37 under drought stress were statistically similar (*p* ˃ 0.05) with control (Figure 9). However, reductions in SOD and POD activities under chilling and drought + chilling stress were higher than those under drought stress. The catalase (CAT) activities were significantly (*p* < 0.05) increased only in XD889 under the individual effect of drought and chilling stresses compared with control. The glutathione reductase (GR) activities tend to increase in XD889, XD319, and Yu37 under all stress treatments; however, significant (*p* < 0.05) differences were only observed for XD319 under chilling and Yu37 under all the stress treatments (Figure 9).

**Figure 10:**
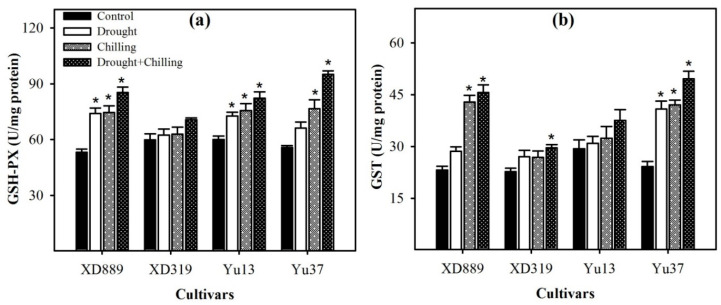
Activities of glutathione peroxidase (**a**) and glutathione S-transferase (**b**) in the leaves of four maize cultivars as influenced by the drought, chilling, and drought + chilling stresses. Vertical bars above mean indicate standard error of three replicates. Mean value for each treatment with * indicate significant differences by the LSD test (*p* ≤ 0.05).

In all maize cultivars, glutathione peroxidase (GSH-PX) and glutathione S-transferase (GST) activities were noticeably increased under stress conditions as compared with control, nonetheless, such enhancement was more by combined drought + chilling stress as compared with their individual effects (Figure 10). Compared with control, all the stress treatments significantly (*p* < 0.05) increased the GSH-PX activities in XD889 and Yu13, and GST activities in Yu37. Whilst, chilling stress and drought + chilling stress recorded significant enhancement in GSH-PX of Yu37, and GST activities of XD889 with respect to the control (Figure 10).

### 2.7. Non-Enzymatic Antioxidants and Total Antioxidant Activity 

**Figure 11:**
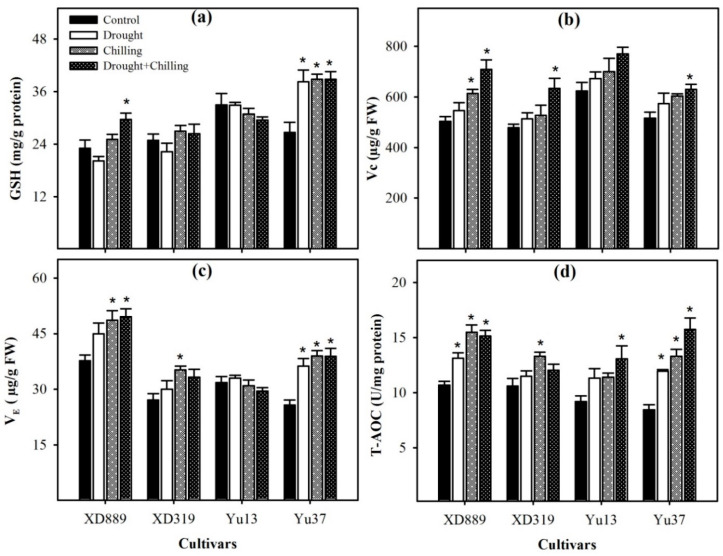
Levels of glutathione content (**a**), vitamin C (**b**), vitamin E (**c**), and total antioxidant capacity (**d**) in the leaves of four maize cultivars as influenced by the drought, chilling, and drought + chilling stresses. Vertical bars above mean indicate standard error of three replicates. Mean value for each treatment with * indicate significant differences compared with control according to LSD test (*p* ≤ 0.05).

Variable response of different maize cultivars was observed regarding the levels of non-enzymatic antioxidants under drought, chilling, and drought + chilling stress conditions (Figure 11). Compared with control, the glutathione (GSH) and vitamin E (V_E_) contents in Y37 were significantly (*p* < 0.05) increased under all the stress treatments. In XD889, the GSH content was significantly (*p* < 0.05) increased under drought + chilling, while V_E_ contents were significantly (*p* < 0.05) enhanced under chilling and drought + chilling, with respect to control. In Yu13, both GSH and V_E_ contents were slightly decreased under stress conditions; however, such effects were statistically non-significant (*p* > 0.05). The highest values for V_E_ were recorded in XD889 compared with all other cultivars. The vitamin C (Vc) contents were significantly (*p* < 0.05) increased in XD889 under chilling and drought + chilling, and in XD319 and Yu37 under drought + chilling, compared with their respective controls. The total antioxidant capability (T-AOC) activity was triggered under stress conditions in all the maize cultivars. Compared with control, the T-AOC activities in XD889 and Yu37 were significantly (*p* < 0.05) increased under all the stress treatments. In XD319 and Yu13, the T-AOC activities were significantly increased under chilling alone and drought + chilling, respectively, compared with their respective controls. Among maize cultivars, XD889 recorded higher T-AOC than XD319, Yu13, or Yu37 (Figure 11d).

### 2.8. Metabolites Accumulation

**Figure 12:**
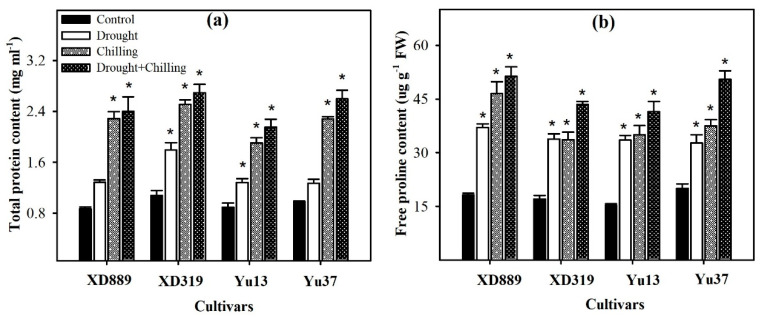
Accumulation of total soluble proteins (**a**) and free proline contents (**b**) in the leaves of four maize cultivars as influenced by the drought, chilling, and drought + chilling stresses. Vertical bars above mean indicate standard error of three replicates. Mean value for each treatment with * indicate significant differences compared with control according to LSD test (*p* ≤ 0.05).

Considerable variations were recorded regarding total soluble protein and free proline contents in maize cultivars under the influence of drought, chilling, and drought + chilling stresses (Figure 12). Compared with control, all the stress treatments significantly increased the total soluble protein and free proline contents in all maize cultivars, only total soluble protein contents in XD889 and Yu37 under drought stress were statistically similar (*p* ˃ 0.05) with their respective controls. The stress-induced enhancements in both these attributes were more under combined occurrence of drought + chilling stresses than those under the individual effect of each stress. Among maize cultivars, XD889 recorded higher free proline contents than XD319, Yu13, or Yu37 (Figure 12).

## 3. Discussion

Water deficit and suboptimal temperature are adverse environmental factors that impose drastic effects on crop plants [2,22]. Maize is comparatively more sensitive to water scarcity and low temperature conditions particularly at the early stages of plant growth [2,25,26,27]. The present study indicated that all the maize shoot growth attributes were severely hampered by drought, chilling, and combination of drought + chilling stresses (Figure 1 and Figure 2). However, the concurrent damage caused by drought and chilling was found to be more severe for all shoot growth attributes than their individual effects. The stress induced reductions in shoot length and biomass were higher under chilling and drought + chilling compared with those under drought stress (Figure 1). Early-season drought coupled with chilling stress limits the seed germination and early stand establishment, possibly due to the reduced intake of water during seed imbibition, and restricted cell division and elongation [2,18,21,28]. Previously, drought and chilling stresses have been reported to decrease the seedling growth by suppressing cell division and elongation, decreasing leaf initiation rate, disturbing the plant water relations, and by causing physiological injuries in plant tissues [1,2,9,18,29,30]. Particularly, cell division and cell elongation are important for leaf area accumulation, while leaf initiation rate is critical for the number of leaves in plants [2,9]. In the present study, greater reductions in shoot growth under chilling and drought + chilling (Figure 1 and Figure 2) might be attributed to decreased cell division and elongation as indicated by significant reductions in leaf width (Figure 3b), and lower leaf initiation rate, which led to decrease in number of leaves under these stresses (Figure 3a). Higher stress-induced reductions in shoot fresh weight compared with shoot length (Figure 1) also indicate that leaf size might have mainly contributed in reduction of shoot fresh weight of maize in the present study.

Root system architecture plays key role in acquisition of water and nutrients, provides structural support, and ensures tolerance against abiotic stresses. It been well reported that the identification of varieties with deeper and more developed root system architecture will help plants to acclimatize with wide range of climatic conditions [2,9]. In the present study, all the stress treatments particularly chilling and drought + chilling hampered the root growth and morphology of all maize cultivars; however, variations in response of maize cultivars to different stress treatments were apparent regarding root growth (Figure 4, Figure 5 and Figure 6). Chilling- and drought + chilling-induced reductions in root growth might be attributed to decrease in root length and biomass (Figure 4), which ultimately decreased the volume and surface area of root system (Figure 6) for exploring the water and nutrients. The XD889 recorded better root growth compared with all other cultivars under control and stress conditions; the root length, total root length, and root diameter of XD889 under stress conditions were statistically similar to the control. A better root system of XD889 (Figure 4, Figure 5 and Figure 6) also ensured the higher aboveground growth (Figure 1, Figure 2 and Figure 3) and greater tolerance of this cultivar under stress conditions. 

In the present study, the chlorophyll contents remained statistically unchanged under chilling stress, compared with the control. Whereas drought stress and drought + chilling stress were found to cause pronounced reductions in total chlorophyll contents and chlorophyll a contents (Figure 7). These reductions might be attributed to reduced water supply and a decrease in leaf water content [31], which ultimately declined the synthesis of photosynthetic pigments in maize plants. 

Both chilling and drought stresses have been reported to cause the oxidative damage in plants by the overproduction of ROS in cells [2,9,18,32,33]. However, plants have developed a complex antioxidative defense system, which consists of non-enzymatic and enzymatic components to overcome the excessive production of ROS [19,26,34,35]. The balance between ROS generation and antioxidants (enzymatic and non-enzymatic) is critical in all plant species under stress conditions [12,29,33,34,36]. Among various antioxidants, the SOD is involved in the dismutation of O_2_^−^ to O_2_ and then H_2_O_2_, whilst CAT, POD, GR and GSH-PX catalyze H_2_O_2_ to H_2_O and O_2_ [11,12,19,36]. In the present study, all the ROS (O^2−^, H_2_O_2_, OH^−^) were significantly enhanced under all the stress treatments, which led to increase in lipid peroxidation rate, as indicated by significantly higher MDA contents under stress conditions (Figure 8). Nevertheless, the differences regarding these attributes were apparent among stress treatments and cultivars. The accumulations of O_2_^−^, H_2_O_2_, and OH^−^ in maize leaves were higher in chilling or drought + chilling compared with drought. This might be possibly due to greater detoxification of ROS under drought stress owing to higher activities of SOD, POD, GSH-PX, GST, and T-AOC compared with chilling or drought + chilling stresses (Figure 9 and Figure 10). A significant increase in O_2_^−^ under stress conditions (Figure 8) might be attributed to inefficiency of SOD to detoxify it, and data (Figure 8) indicate that reductions in SOD activity under stress conditions were well concomitant with enhanced accumulation of O^2−^. Likewise, the trend of increase in H_2_O_2_ accumulation was consistent with decrease in POD activity under stress conditions (Figure 8 and Figure 9). The CAT activity in XD889 was only increased under the individual effects of chilling or drought stress, and such effects were not observed under combined stresses (Figure 9). Recently, some reports proved that the extreme stress conditions could imbalance the active oxygen metabolism as well as antioxidant enzyme activities [17,37,38]. Cao et al. (2011) found that the SOD and POD activities were initially increased under drought and low temperature conditions and were decreased later with their prolonged exposure in oil palm owing to the severity of stress [37].

Non-enzymatic antioxidants like GSH, Vc, and V_E_ have also been reported to play key role in stress tolerance of crop plants [11,19]. The GSH can directly detoxify the O_2_^−^, OH^−^,and H_2_O_2_ and; therefore, contributes in stress tolerance in plants [17,18,35]. Hussain et al. (2016) reported that the Vc and V_E_ are involved in various metabolic processes as non-enzymatic scavenger to quench ROS under stress conditions [19]. In the present study, GSH and V_E_ contents were increased in XD889, XD319, and Yu37 under chilling and drought + chilling stresses, but slightly decreased in Yu13. The response of GSH in different rice cultivars (Figure 11) was well concomitant with the GR activity under different stress treatments (Figure 10). The T-AOC in maize seedlings, particularly in XD889, was up-regulated by drought, chilling, and drought + chilling stress conditions (Figure 11), nevertheless, the stress-induced oxidative damage in the present study indicates that the levels/activities of antioxidants were not enough to overcome the excessive ROS production. The XD889 was found to record higher T-AOC, SOD, POD, CAT, and GR activities compared with other maize cultivars under stress conditions (Figure 9, Figure 10 and Figure 11), which is consistent with lower oxidative damage (MDA contents) (Figure 8) and better above- and below-ground growth of this cultivar (Figure 1, Figure 3, Figure 4, and Figure 6). 

Drought, chilling, and drought + chilling stresses triggered the accumulation of total soluble protein and free proline contents in all maize cultivars (Figure 12). The stress-induced enhancements in both these attributes were more under combined drought + chilling stress, which might be the response of maize seedlings to the severity of stress. These compatible solutes are known to protect the plants from osmotic stress by not posing any detrimental effects on enzymes, membranes, and other macromolecules even at higher concentrations [2,9]. Krasensky and Jonak (2012) stated that the proline, carbohydrates, and proteins are generally involved in maintaining cell turgor by osmotic adjustment, and sustaining redox metabolism to quench ROS [14], and accumulation of these metabolites is triggered under stress conditions [17]. Higher concentrations of proline were also found in oil palm under drought and low temperature stresses [37]. In the present study, XD889 recorded higher free proline contents than all other cultivars (Figure 12), which might have helped in maintenance of plant water status and regulation of stress tolerance in this cultivar particularly against drought. 

The southwest region (District Beibei, Chongqing) of China is colder in mid-Feb to mid-March and hotter in the end of June to the end of August. Thus, early-cultivated maize in field conditions faces the sub-optimal soil temperature, which reduces the germination rate and seedling establishment of the crop. On the other hand, if a crop is planted late in the season, it faces high temperatures in the reproductive stages in the field, which hampers the grain filling and productivity of maize. Therefore, the results of the present study might be of great interest for the maize farmers in the southwest region of China. 

## 4. Materials and Methods

### 4.1. Plant Material, Growth Conditions, and Treatments 

This experiment was conducted in the controlled growth chamber by using hydroponic solution at the College of Agronomy and Biotechnology (CAB), Southwest University (SWU), Chongqing, China (longitude 106°26′02″ E, latitude 29°49′32″ N, and altitude 220 m) during spring 2016. The seeds of two maize hybrids (i.e., Xida889 (XD889) and Xida319 (XD319)) and two maize inbred (i.e., Yu13 and Yu37) were obtained from Maize Research Institute, CAB, SWU, Chongqing, China. The XD889 and XD319 are the widely cultivated middle-early maize hybrids which are appropriate to cultivate early in the season in the southwest area of China. In our previous investigations, XD889 appeared comparatively more tolerant to drought stress compared with XD319 [32]. Whilst, the Yu13 and Yu37 are the advanced inbred lines of Maize Research Institute, CAB, SWU, China. The initial germination rate and moisture (on dry weight basis) content of the seeds were >90% and <10%, respectively. Prior to sowing, seeds were surface sterilized with NaOCl solution to minimize contamination and rinsed thrice with sterile distilled water. 

The maize seed were kept on wet muslin cloth in normal condition for germination. After germination (7 days after sowing; DAS), seedlings were transplanted to plastic pots (30 × 20 × 12 cm) filled with modified Hoagland’s nutrient solutions [39]. Plastic pots containing 4 L of respective solution and a floating board with four separated sections (for maize cultivars) were used. Six seeds of each cultivar were sown. Six plants per cultivar were transplanted in each section of the board. The experiment was laid out in a completely randomized design with three replicates.

The maize seedlings were subjected to drought and/or low temperature stresses after four days of transplanting. All the pots were placed in a growth chamber and the temperature was set to 25 and 20 °C with 12:12 h light:dark period for control and drought stress treatments, and temperature was set to 15 and 12 °C with 12:12 h light:dark period for chilling and chilling + drought stress treatments. The light intensity was 25,000 Lx and the air humidity during the course of the study was maintained at 75% in all growth chambers. Polyethylene glycol (PEG) has a high molecular weight, thus drought stress was imposed by using 15% PEG-MW6000 solution corresponding to final osmotic potentials of −0.51 MPa. The combined stress consisted of simultaneous treatment with PEG and chilling stress. The drought and chilling stress treatments were selected based on the results of various preliminary studies (data not shown). Nutrient solution with or without PEG (15%) was changed after every 4 days. After 23 DAS, seedlings were harvested and growth parameters were recorded, whereas fresh maize seedlings were stored at −80 °C for measuring different biochemical analysis. 

### 4.2. Observations

#### 4.2.1. Measurement of Growth Parameters

Shoot and root length of maize seedlings were recorded with a meter scale, whereas an electronic weighing balance was used to measure the shoot and root fresh biomass. The maximum leaf width and stem diameter were measured with measuring tape and Digimatic caliper (500-197-30, Mitutoyo group, Kanagawa, Japan), while the number of leaves was counted manually. Total root length, root surface area, average diameter, and root volume were analyzed with WinRHIZO by using a Epson Perfection V700 Photo Flatbed scanner (No. B11B178023, PT Epson, Jakarta, Indonesia).

#### 4.2.2. Photosynthetic Pigments

Chlorophyll (Chl a, Chl b, and total Chl) concentrations were determined according to the Peng and Liu (1992) method [40]. Extraction of 250 mg leaf without vein (leaf blade) was done with 10 mL ethanol-acetone (vol. 1:2), and the extract was transferred to a 15 mL tube. The tubes were placed in dark to avoid light for 24 h. The absorbance was measured at 645, 663, and 652 nm. The chlorophyll content was computed by the following formulae: 

For Chlorophyll a content (mg/g tissue) = (12.7D_663_ − 2.69D_645_) × V/(1000 × W)

For Chlorophyll b Content (mg/g tissue) = (22.7 D_645_ − 4.68D_663_) × V/(1000 × W)

For Total Chlorophyll (mg/g tissue) = D_652_ × V/(34.5×W)/Chl a + Chl b

Where D663, D645, and D652, respectively, are the corresponding wavelengths of the light density value, V is extracting liquid volume, and W is leaf fresh weight.

#### 4.2.3. Lipid Peroxidation and ROS Activity 

Lipid peroxidation was determined as malondialdehyde (MDA) content, measured by thiobarbituric (TBA) method using the “MDA Detection Kit (A003)” purchased from Nanjing Jiancheng Bioengineering Institute, Nanjing, China (www.njjcbio.com). The absorbance for MDA was measured at 532 nm and expressed as nmol/g fresh weight. 

The contents of hydrogen peroxide (H_2_O_2_), hydroxyl free radical (OH^−^), and superoxide anion radical (O_2_^−^) in the leaves of maize seedlings were determined using the commercial kits viz., “H_2_O_2_ Detection Kit (A064)”, “OH^−^ Detection kit (A018)”, and “O_2_^−^ Detection kit (A052)”, respectively, obtained from Nanjing Jiancheng Bioengineering Institute, Nanjing, China according to the manufacturer’s instructions. The H_2_O_2_ bound with molybdic acid to form a complex, which was measured at 405 nm and the content of H_2_O_2_ was then calculated. The OH^−^ was expressed as unit mg^−1^ protein, and one unit was the amount required to reduce 1 mmol/L of H_2_O_2_ in the reaction mixture per minute at 37 °C. The O_2_^−^ inhibition per g tissue protein for 40 min in 37 °C reaction which equals to superoxide anion radical inhibition caused by 1mg Vc is considered as 1 anti-superoxide anion radical activity unit.

#### 4.2.4. Estimation of Enzymatic and Non-Enzymatic Antioxidants

The activities of enzymatic antioxidants were detected by using the commercial kits in accordance with the manufacturer’s instructions. The kits for superoxide dismutase (A001), peroxidase (A084-3), catalase (A007-2), glutathione peroxidase (A005), glutathione reductase (A062), and glutathione S-transferase (A004) were purchased from the same company as mentioned above. The absorbance readings of SOD, POD, CAT, GPX, GR, and GST were detected at 550, 420, 405, 412, 340, and 412 nm, respectively (Tecan-infinite M200, Switzerland). The SOD, POD, CAT, GPX, and GST activities were expressed as units U/mg protein, while GR activity was demonstrated as units U/g protein. The units of the antioxidant enzyme activities were defined as follows: One unit of SOD activity was the amount of enzyme required to decrease the reference rate to 50% of maximum inhibition; one unit of POD activity was defined as the amount of enzyme necessary for the decomposition of 1 µg substrate in 1 min at 37 °C; one unit of CAT activity was defined as the amount of enzyme required to decompose the 1 µM H_2_O_2_ in 1 s at 37 °C; one unit of GPX activity was the amount of enzyme required to oxidize 1 µM GSH in 1 min at 37 °C; one unit of GR activity was defined as the amount of enzyme depleting 1 mM NADPH in 1 min; and one unit of GST activity was defined as the amount of enzyme depleting 1 µM GSH in 1 min.

The glutathione (GSH), ascorbic acid (Vitamin C), and vitamin E content in leaves of maize seedlings were measured by colorimetric method using the “GSH (deproteinization) assay kit-A006”, “Vitamin C assay kit-A009”, and “Vitamin E assay kit-A008”, respectively, purchased from the Nanjing Jiancheng Bioengineering Institute, Nanjing, China. The absorbance for GSH, Vc, and V_E_ were recorded at 420 nm, 536 nm, and 533 nm (Tecan-infinite M200, Tecan Trading AG, Männedorf, Switzerland), respectively. The GSH content was expressed as mg/g protein, and the Vc and V_E_ expressed as µg g^−1^ tissue fresh weight. The commercial “kit-A015” (Nanjing Jiancheng Bioengineering Institute, Nanjing, China) was used for determination of total antioxidant capability (T-AOC). The absorbance for T-AOC was measured at 520 nm and data were expressed as U/mg protein. One unit of T-AOC was defined as the amount that increased the OD value per mg tissue protein per minute by 0.01 reaction system at 37 °C.

#### 4.2.5. Assay of Osmolyte Accumulation Profiles

Free proline (FP) contents were assessed by following the method of Shan et al. (2007) [41]. Briefly, fresh leaves (0.5 g) sample was extracted with 5 mL of 3% sulfosalicylic acid at 100 °C for 10 min with shaking. The extracts were filtered through glass wool and analyzed for proline content using the acid ninhydrin method, and the absorbance was read at 520 nm. The resulting values were compared with a standard curve constructed using known amounts of proline (Sigma, St. Louis, MO, USA).

The content of total protein in the leaves of maize seedling was determined by Coomassie brilliant blue method using the “Total Protein Quantification Kit (A045-2)” obtained from Nanjing Jiancheng Bioengineering Institute, China, and unit of protein was quantified as mg ml^−1^ sample solution. 

### 4.3. Statistical Analysis

The data collected were statistically analyzed following the analysis of variance technique using Statistix 8.1 (Analytical Software, Tallahassee, FL, USA) software and the mean variance of the data was analyzed using the least significant difference (LSD) test at the 0.05 probability level. Sigma Plot 12.5 (Systat Software Inc., San Jose, CA, USA) was used for graphical presentation of the data.

## 5. Conclusions

Individual and concurrent exposure of drought and chilling stresses hampered the morpho-physiological growth and oxidative status of maize cultivars, nevertheless, the interactive damage caused by drought + chilling was found to be severe for all the studied traits. Between two individual stress factors, chilling-induced reductions in seedling length and biomass of maize cultivars were more compared with drought stress alone. Greater decrease in root length and biomass under chilling stress ultimately decreased the volume and surface area of the root system, and restricted the shoot growth. All the stress treatments, particularly chilling and drought + chilling, triggered oxidative stress by higher ROS accumulation and rate of lipid peroxidation. Variations in response of maize cultivars were also apparent against different stress treatments, and XD889 performed comparatively better than the rest of the cultivars. The better growth and greater stress tolerance of this cultivar was attributed to vigorous root system architecture, as indicated by higher root biomass, root surface area, and root volume under drought and chilling stresses. Moreover, an efficient antioxidant defense system in terms of higher T-AOC, SOD, POD, CAT, and GR activities also contributed in greater stress tolerance of XD889 over other cultivars.
